# Higher Body Mass Index Increases the Risk for Biopsy-Mediated Detection of Prostate Cancer in Chinese Men

**DOI:** 10.1371/journal.pone.0124668

**Published:** 2015-04-10

**Authors:** Meng-Bo Hu, Pei-De Bai, Yi-Shuo Wu, Li-Min Zhang, Hua Xu, Rong Na, Hao-Wen Jiang, Qiang Ding

**Affiliations:** Department of Urology, Huashan Hospital, Fudan University, Shanghai, China; McMaster University, CANADA

## Abstract

**Objective:**

To investigate the relationship between body mass index (BMI) and prostate cancer (PCa) risk at biopsy in Chinese men.

**Patients and Methods:**

We retrospectively reviewed the records of 1,807 consecutive men who underwent initial multicore (≥10) prostate biopsy under transrectal ultrasound guidance between Dec 2004 and Feb 2014. BMI was categorised based on the Asian classification of obesity as follows: <18.5 (underweight), 18.5–22.9 (normal weight), 23–24.9 (overweight), 25–29.9 (moderately obese), and ≥30 kg/m2 (severely obese). The odds ratios (OR) of each BMI category for risk of PCa and high-grade prostate cancer (HGPCa, Gleason score ≥4+3) detection were estimated in crude, age-adjusted and multivariate-adjusted models. Prevalence ratios and accuracies of PSA predicted PCa were also estimated across BMI groups.

**Results:**

In total, PCa was detected by biopsy in 750 (45.4%) men, and HGPCa was detected in 419 (25.4%) men. Compared with men of normal weight, underweight men and obese men were older and had higher prostate specific antigen levels. The risk of overall PCa detection via biopsy presented an obvious U-shaped relationship with BMI in crude analysis. Overall, 50.0%, 37.4%, 45.6% 54.4% and 74.1% of the men in the underweight, normal weight, overweight, moderately obese and severely obese groups, respectively, were diagnosed with PCa via biopsy. In multivariate analysis, obesity was significantly correlated with a higher risk of PCa detection (OR = 1.17, 95%CI 1.10–1.25, P<0.001). However, higher BMI was not correlated with HGPCa detection (OR = 1.03, 95%CI 0.97–1.09, P = 0.29). There were no significant differences in the accuracy of using PSA to predict PCa or HGPCa detection across different BMI categories.

**Conclusion:**

Obesity was associated with higher risk of PCa detection in the present Chinese biopsy population. No significant association was detected between obesity and HGPCa.

## Introduction

Obesity, a global public health concern, has been repeatedly linked to the development of different cancers in epidemiologic and basic research studies [1,2]. In prostate cancer (PCa), which claims more than a quarter of a million deaths worldwide annually [3], progression and prognosis have been shown in various studies in Western populations to be inversely correlated with the patient’s body mass index (BMI) [4,5]. Our previous meta-analysis revealed that BMI was associated with a 15% and 37% higher risk of PCa detection and high-grade prostate cancer (HGPCa) detection, respectively, at biopsy [6].

In the past few decades, most Asian countries have experienced a boom in the incidence and mortality rate of PCa [7], which has been generally attributed to a Westernised life style, elevated body weight and increased prostate biopsy rates in Asian populations. Meanwhile, previous studies in Asian populations have drawn contradictory conclusions that BMI had positive, null or negative impacts on the risks of PCa and HGPCa [8–11]. Recently, Masuda et al. reported higher rates of PCa and HGPCa detection in patients with a BMI <21 kg/m^2^ compared to those with a BMI from 21–22.9 kg/m^2^ in Japanese men [12]. A U-shaped relationship between BMI and mortality from all cancers in age- and smoking-adjusted analyses was also detected in a large Asia-Pacific cohort study [13]. In order to explore the relationship between BMI and biopsy-mediated PCa and HGPCa detection in Chinese population, whose BMI distributions was quite different from Western men, we performed a cross-sectional study of BMI and the risk of PCa and HGPCa at biopsy. The significance of this research could be extensive, as an intention to reveal the real impact of BMI on biopsy outcomes might be helpful for optimising the current PCa screening strategies for Chinese populations with different body weights.

## Patients and Methods

### Study population and study variables

After obtaining the approval of institutional review board of Huashan Hospital, we retrospectively reviewed the clinical records of 1,807 consecutive men who underwent initial multicore (≥10) prostate biopsy between Dec 2004 and Feb 2014 at a tertiary referral hospital in Shanghai, China. Written informed consent was obtained from patients for their clinical records to be used in this study before the biopsy procedure. Biopsy indications included the following: (1) elevated PSA levels (>4 ng/ml); (2) normal PSA levels with a suspicious free to total PSA ratio (fPSA/tPSA) <0.16 or PSA density (PSAD) >0.15; and (3) positive findings from digital rectal examination (DRE), transrectal ultrasound (TRUS) or magnetic resonance imaging (MRI). All multicore needle biopsies were performed under TRUS guidance. Patients with unavailable BMI data (N = 134) and those from non-Asian racial backgrounds (N = 22) were excluded from the study, resulting in a final study population of 1651 patients.

Data on age, pre-biopsy BMI, pre-treatment PSA and fPSA, prostate volume (PV), DRE findings, diagnostic imaging findings and pathological outcomes of prostate biopsy were collected retrospectively by reviewing the patients’ medical charts. BMI (kg/m^2^) was calculated as weight in kilograms divided by height in meters squared. All men were categorised into five groups according to BMI, calculated on the basis of the criteria and classification of obesity in Asia [14] as follows: underweight (<18.5 kg/m^2^), normal weight (18.5–22.9 kg/m^2^), overweight (23–24.9 kg/m^2^), moderately obese (25–29.9 kg/m^2^) and severely obese (≥30 kg/m^2^). Biopsy specimens containing adenocarcinoma were scored according to the Gleason grading system, and HGPCa was defined as the presence of a Gleason score ≥4+3.

### Statistical analysis

Differences in patient characteristics (age, BMI, PSA, %fPSA, PSAD, PV, DRE and TRUS findings), overall PCa detection rate and HGPCa detection rate across BMI categories were compared using the Kruskal-Wallis test for continuous variables and the chi-squared test for categorical variables. To examine the association between categorical or continuous BMI and biopsy-mediated detection of PCa and HGPCa, we carried out crude, age-adjusted and multivariate analysis. In the multivariate logistic regression model, we adjusted for age (continuous), PSA (continuous), %fPSA (continuous), PV (continuous), DRE findings (abnormal versus normal) and TRUS findings (hypoechoic nodules versus normal). PSA, %fPSA and PV were analysed after logarithmic transformations in multivariate analysis because these variables were not normally distributed. Prevalence ratios were calculated across different BMI groups [15]. According to the different BMI categories, the accuracies of PSA in predicting PCa and HGPCa in each group were assessed by the area under curve (AUC) of the receiver operating characteristics (ROC) curves. The statistical significance of the differences between various predictive accuracy estimates was compared using chi-squared tests.

Statistical analyses were performed using SPSS 20 (SPSS, Chicago, IL, USA) and STATA/SE 12 software (StataCorp, College Station, TX, USA). A two-tailed P value <0.05 was considered to be statistically significant in all analyses.

## Results

### Patient characteristics

The baseline characteristics of the overall biopsy population are presented in Table 1. For the 1,651 men enrolled in the present study, the median age was 72 years, the median BMI was 23.2 kg/m^2^, the median PSA level was 15.7 ng/ml, the median PV was 49 ml, the median %fPSA was 14, and the median PSAD was 0.31. A total of 31.6% of the biopsy population had positive DRE findings, and 54.0% of these patients presented hypoechoic TRUS nodules. In total, PCa was detected via biopsy in 750 (45.4%) men, and HGPCa was detected in 419 (25.4%) men.

**Table 1:** Clinical characteristics and biopsy outcomes of the study population stratified by BMI.

Variables	Total	Underweight	Normal weight	Overweight	Moderately obese	Severely obese	P-value
		(BMI <18.5 kg/m^2^)	(BMI 18.5–22.9 kg/m^2^)	(BMI 23–24.9 kg/m^2^)	(BMI 25–29.9 kg/m^2^)	(BMI ≥30 kg/m^2^)	
**No. patients (%)**	1651	94 (5.7%)	661 (40.0%)	419 (25.4%)	450 (27.3%)	27 (1.6%)	-
**BMI** *, **kg/m** ^**2**^	23.2 (4.1)	17.6 (1.2)	21.3 (1.9)	23.9 (0.9)	26.4 (2.0)	31.0 (1.2)	<0.001^†^
**Age** *, **years**	72 (13)	76 (9)	73 (12)	71 (12)	71 (13)	73 (17)	<0.001^†^
**PSA** *, **ng/ml**	15.7 (29.7)	21.4 (38.3)	13.9 (24.3)	14.0 (19.5)	17.4 (33.9)	17.3 (87.3)	0.004^†^
**PV** *, **ml**	49 (31.2)	43.5 (32.5)	51.0 (31.0)	50.0 (29.0)	49 (33.0)	47 (30.3)	0.542^†^
**%fPSA** *	14 (11)	13 (12)	15 (11)	14 (11)	13 (10)	11.5 (8)	0.051^†^
**PSAD** *	0.31 (0.74)	0.47 (1.25)	0.28 (0.7)	0.28 (0.63)	0.35 (0.89)	0.47 (1.31)	0.005^†^
**Abnormal DRE, n/N (%)**	504/1594 (31.6%)	33/94 (35.1%)	183/637 (28.7%)	112/407 (27.5%)	163/430 (37.9%)	13/25 (52.0%)	0.001^‡^
**Hypoechoic TRUS nodule, n/N (%)**	845/1565 (54.0%)	46/88 (52.3%)	335/631 (53.1%)	204/396 (51.5%)	245/428 (57.2%)	15/22 (68.2%)	0.298^‡^
**PCa detected from biopsy, n (%)**	750 (45.4%)	47 (50.0%)	247 (37.4%)	191 (45.6%)	245 (54.4%)	20 (74.1%)	<0.001^‡^
**D’Amico risk**							0.007
** Low**	35 (4.7%)	1 (2.1%)	10 (4.0%)	8 (4.2%)	14 (5.7%)	2 (10.0%)	
** Intermediate**	154 (20.5%)	2 (4.3%)	40 (16.2%)	53 (27.7%)	54 (22.0%)	5 (25.0%)	
** High**	561 (74.8%)	44 (93.6%)	197 (79.8%)	130 (68.1%)	177 (72.2%)	13 (65.0%)	
**HGPCa detected from biopsy, n (%)**	419 (25.4%)	23 (24.7%)	160 (24.2%)	91 (21.9%)	136 (30.4%)	9 (33.3%)	0.042^‡^

*BMI* body mass index, *PSA* prostate specific antigen, *%fPSA* free PSA/total PSA (%), *PSAD* PSA density, *PV* prostate volume, *DRE* digital rectal examination, *TRUS* transrectal ultrasound, *PCa* prostate cancer, *HGPCa* high-grade prostate cancer

*Continuous variables are shown as the median value and interquartile range

^†^Using the Kruskal-Wallis test

^‡^Using the chi-squared test

**Fig 1:**
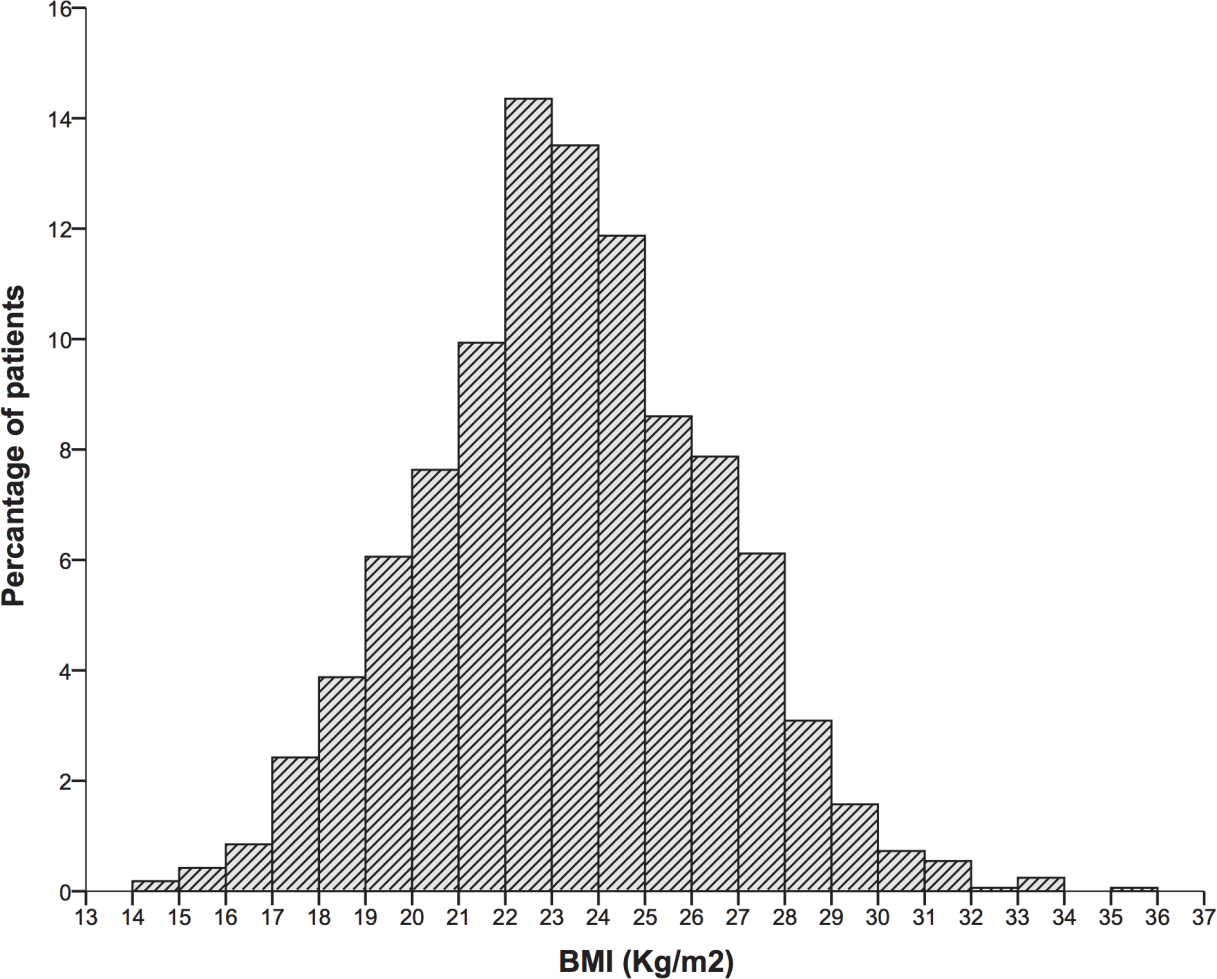
Distribution of BMI in the study population.

Fig 1 displays the distribution of BMI in the overall study population. When the aforementioned Asian criteria of BMI categories was applied, the numbers of patients with a BMI <18.5 kg/m^2^, 18.5–22.9 kg/m^2^, 23–24.9 kg/m^2^, 25–29.9 kg/m^2^, and ≥30 kg/m^2^ were 94 (5.7%), 661 (40.0%), 419 (25.4%), 450 (27.3%), and 27 (1.6%), respectively (Table 1). Variables, such as age (P<0.001), PSA (P = 0.004), PSAD (P = 0.005) and abnormal DRE findings (P = 0.001), were significantly different across the BMI groups. Compared with men of normal weight, underweight men and obese men were older, had higher PSA levels, increased PSAD and a greater percentage of positive DRE findings. However, almost no differences in clinical variables were detected between overweight and normal-weight men. Meanwhile, there seemed to be no statistically significant differences in PV, %fPSA and positive TRUS findings across BMI groups.

### BMI and PCa detection

**Fig 2:**
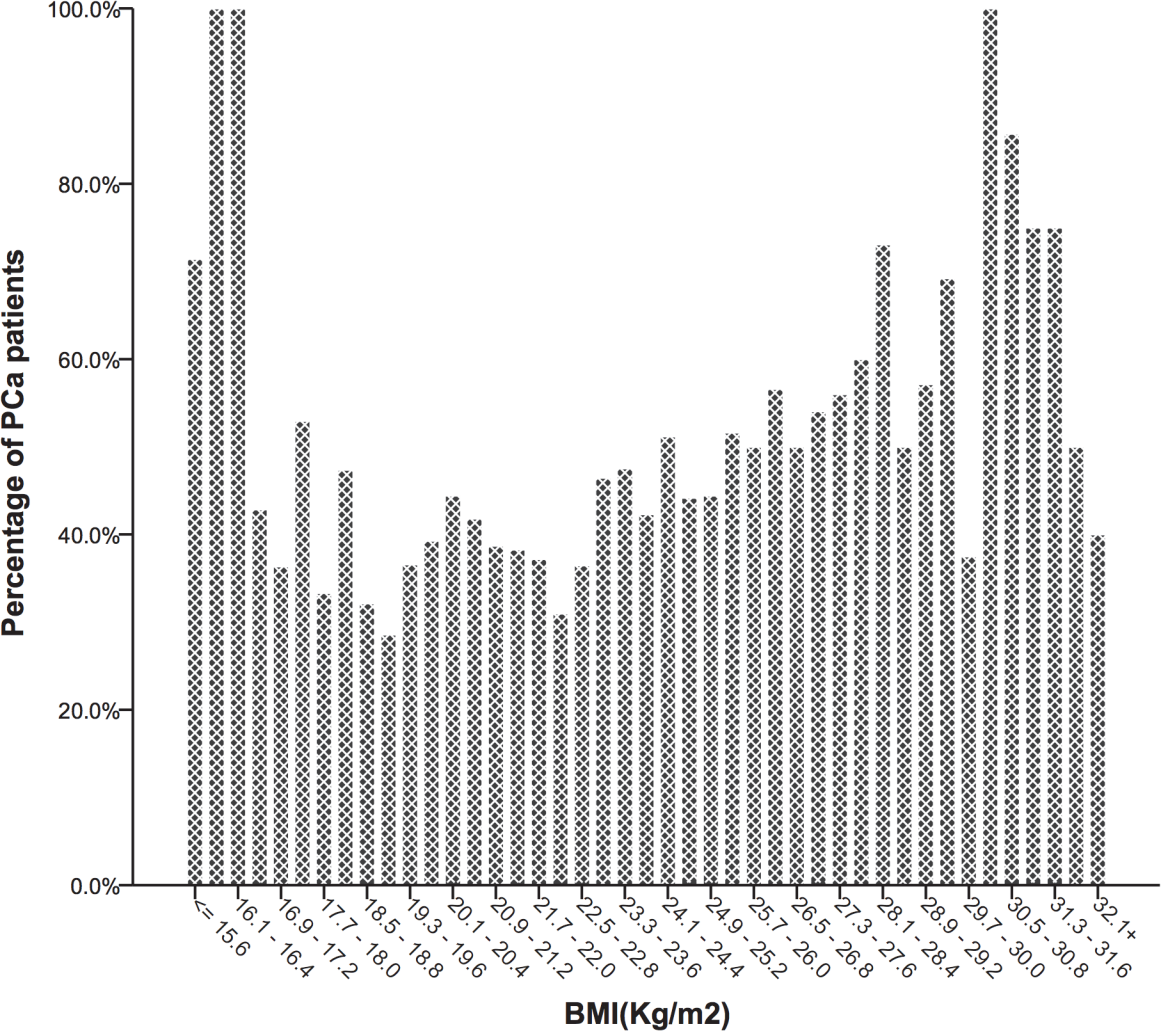
Different PCa detection rates at biopsy, according to BMI distribution.

The risk of overall PCa detection via biopsy presented an obvious U-shaped relationship across BMI categories (Fig 2). There were 50.0%, 37.4%, 45.6%, 54.4% and 74.1% of men diagnosed with PCa via biopsy in the underweight, normal weight, overweight, moderately obese and severely obese groups, respectively (Table 1). 93.6% of underweight PCa patients were in high-risk group, while 72.2% of moderately obese PCa patients and 65.0% of severely obese PCa patients were in high-risk group. There was also an increasing trend of PCa detection in men with a BMI ≥18.5 kg/m^2^, while the trend was inverted when the BMI was lower than 18.5 kg/m^2^. The crude logistic regression analysis and prevalence ratios showed an increased risk in the four study groups compared with the reference group (Table 2). After adjusting for age and all confounding variables, the odds ratio (OR) of being diagnosed with PCa continued to rise in men with a BMI >23 kg/m^2^. On the contrary, the increased risk of PCa detection diminished in the underweight group. For BMI as a continuous variable, obesity was significantly correlated with a higher risk of PCa detection (OR = 1.17, 95%CI 1.10–1.25, P<0.001).

**Table 2:** OR, 95% CI and prevalence ratios of PCa and HGPCa detection at prostate biopsy across BMI groups.

	Categorical BMI (kg/m^2^)					P-value	Continuous BMI	P-value
	<18.5	18.5–22.9	23–24.9	25–29.9	≥30			
	OR (95% CI)	OR (95% CI)	OR (95% CI)	OR (95% CI)	OR (95% CI)		OR (95% CI)	
**PCa detection**								
** Crude**	1.68(1.09–2.59)	1.00(Referent)	1.40(1.10–1.80)	2.00(1.57–2.56)	4.79(2.00–11.49)	<0.001	1.08(1.05–1.12)	<0.001
** Age-adjusted**	1.46(0.94–2.28)	1.00(Referent)	1.50(1.16–1.94)	2.20(1.71–2.83)	4.90(2.01–11.94)	<0.001	1.10(1.07–1.14)	<0.001
** Multivariate-adjusted** ^**†**^	1.04(0.45–2.39)	1.00(Referent)	2.40(1.48–3.90)	3.52(2.22–5.57)	4.10(0.91–18.57)	<0.001	1.17(1.10–1.25)	<0.001
** Prevalence ratios** ^‡^	1.34	1.00(Referent)	1.22	1.46	1.98	-	-	-
**HGPCa detection**								
** Crude**	1.03(0.62–1.70)	1.00(Referent)	0.88(0.65–1.17)	1.36(1.04–1.78)	1.56(0.69–3.55)	0.043	1.03(1.00–1.07)	0.078
** Age-adjusted**	0.94(0.56–1.55)	1.00(Referent)	0.90(0.67–1.21)	1.42(1.08–1.86)	1.53(0.69–3.49)	0.028	1.04(1.01–1.08)	0.022
** Multivariate-adjusted** ^**†**^	0.57(0.25–1.27)	1.00(Referent)	0.91(0.57–1.45)	1.16(0.75–1.78)	1.01(0.30–3.42)	0.53	1.03(0.97–1.09)	0.29
** Prevalence ratios** ^‡^	1.01	1.00(Referent)	0.90	1.25	1.38	-	-	-

*BMI* body mass index, *OR* odds ratio, *CI* confidence interval, *PCa* prostate cancer, *HGPCa* high-grade prostate cancer, *PSA* prostate specific antigen, *%fPSA* free PSA/total PSA (%), *PV* prostate volume, *DRE* digital rectal examination, *TRUS* transrectal ultrasound

^†^Adjusted for age, PSA, %fPSA, PV, DRE, and hypoechoic TRUS nodule

^‡^Prevalence ratios = (Probability of outcome in exposed group)/(Probability of outcome in reference group)

### BMI and HGPCa detection

**Fig 3:**
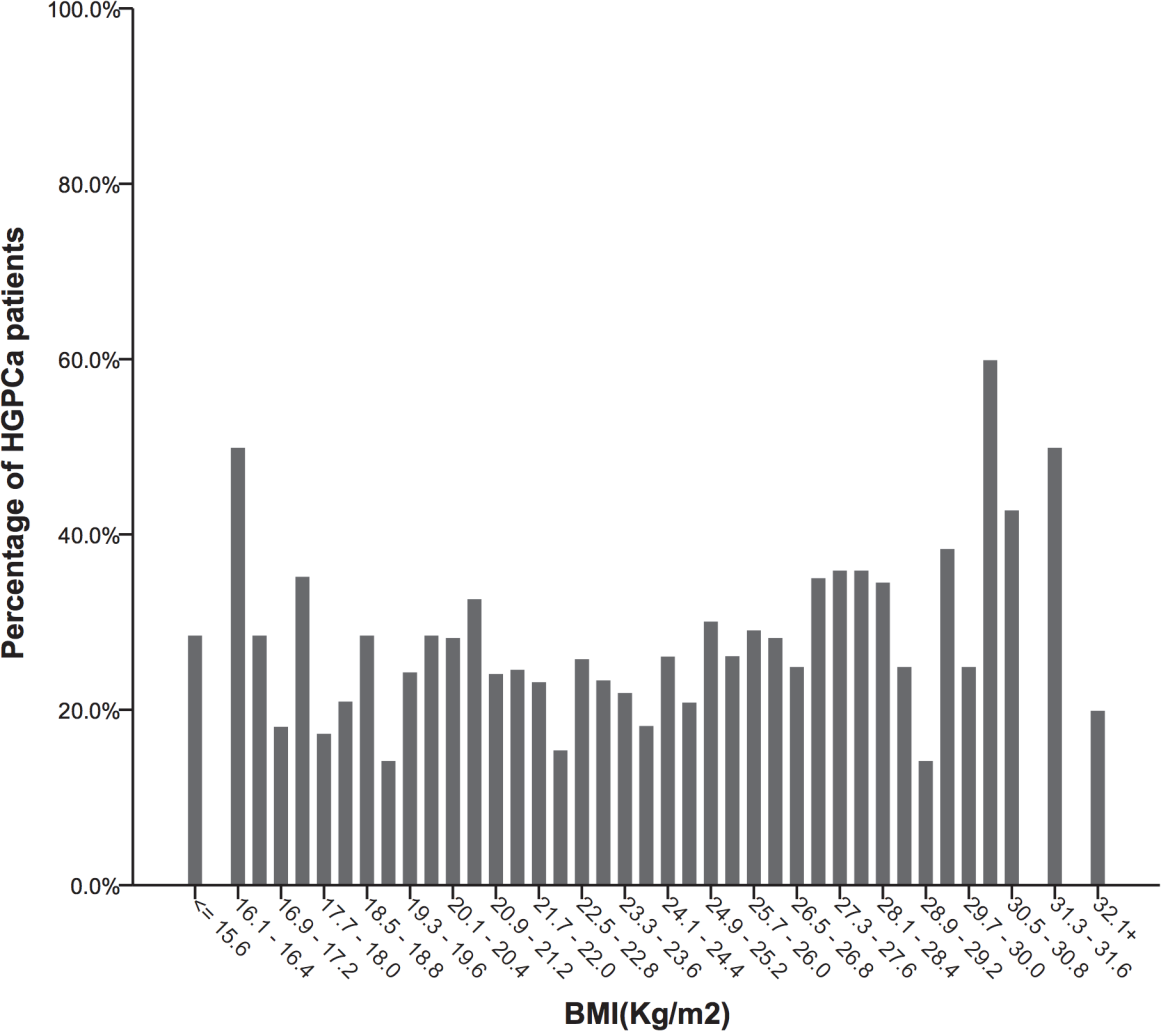
Different HGPCa (Gleason score ≥4+3) detection rates at biopsy, according to BMI distribution.

The risk of HGPCa detection via biopsy presented a much more vague, if any, U-shaped relationship with different BMIs (Fig 3). Overweight men presented the lowest risk of harbouring HGPCa (21.9%). The risks in underweight (24.7%) and normal weight (24.2%) men were slightly higher, and the risks rose steadily in moderately (30.4%) and severely obese (33.3%) men (Table 1). In the logistic regression model, higher BMI (especially BMI >25 kg/m^2^) was correlated with a higher risk of HGPCa detection in the age-adjusted model (OR 1.04, 95%CI 1.01–1.08, P = 0.022) (Table 2). When all confounders were adjusted, these significant positive correlations vanished, and no association of statistical significance was found between BMI and HGPCa detection (OR 1.03, 95%CI 0.97–1.09, P = 0.29). Prevalence ratios were also presented across different BMI groups.

### Predictive accuracy of PSA stratified by BMI categories

**Fig 4:**
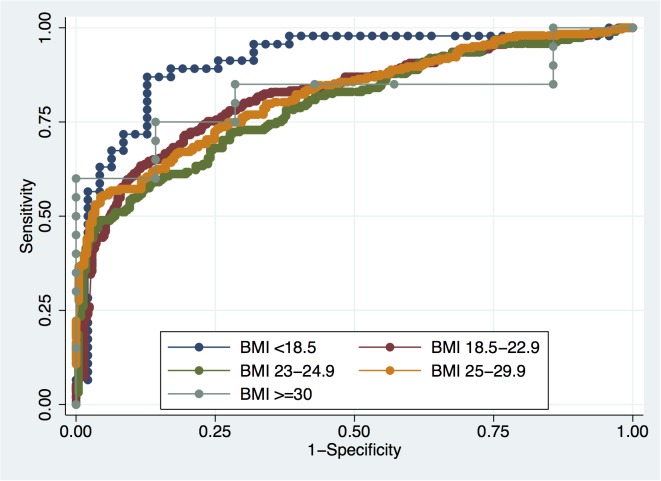
ROC curves for PSA in predicting PCa across BMI categories.

**Fig 5:**
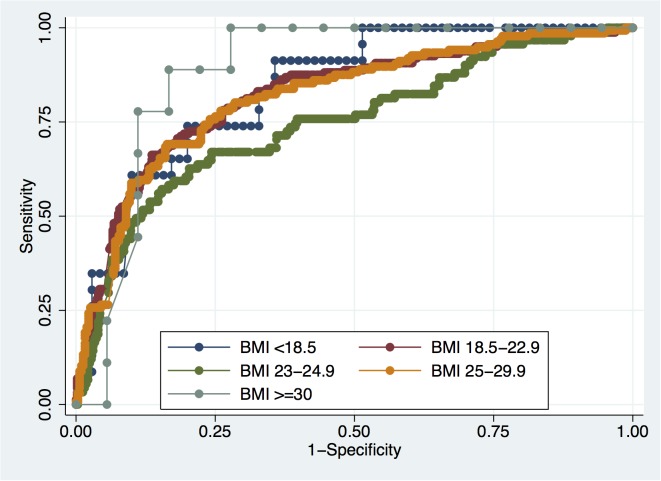
ROC curves for PSA in predicting HGPCa across BMI categories.

The accuracies of PSA in predicting PCa and HGPCa among all patients were 81.9% and 80.9%, respectively, as shown in Table 3. PSA accuracies for PCa detection in each BMI category were 92.5%, 83.1%, 79.7%, 81.2% and 81.2%, respectively (Fig 4). Meanwhile, PSA accuracies for HGPCa in each BMI category were 85.3%, 82.5%, 74.9%, 81.2% and 93.5%, respectively (Fig 5). There were no significant differences in PSA accuracy among the different BMI categories.

**Table 3:** Accuracy of PSA in predicting the detection of PCa and HGPCa at biopsy across BMI categories.

BMI (kg/m^2^)	PCa		HGPCa	
	AUC	95% CI	AUC	95% CI
**Total**	0.816	0.795–0.837	0.805	0.780–0.830
**<18.5**	0.908	0.844–0.973	0.838	0.753–0.923
**18.5–22.9**	0.82	0.785–0.855	0.821	0.783–0.860
**23–24.9**	0.795	0.751–0.838	0.746	0.686–0.806
**25–29.9**	0.821	0.783–0.859	0.815	0.772–0.859
**≥ 30**	0.821	0.659–0.984	0.883	0.749–1.000
**P-value** ^**†**^	0.081		0.182	

*BMI* body mass index, *PCa* prostate cancer, *HGPCa* high-grade prostate cancer, *AUC* area under curve, *CI* confidence interval

^†^Using the chi-squared test

## Discussion

The impact of body weight on PCa detection remains a global health concern. With the increase in the rates of obesity and PCa in Asia, several studies addressing the correlation between BMI and PCa detection among Asian populations have been conducted [8,12,16–18]. Most of previous studies have placed emphasis on the overweight and obese population, categorising the population with a BMI <23 kg/m^2^ as the normal control group. In the present study, considering the left-shifted BMI distribution in Asian populations, compared with Western counterparts, we added a new category (underweight; BMI <18.5 kg/m^2^) and modified the normal-weight category (BMI 18.5–22.9 kg/m^2^) according to the criteria and classification of obesity in Asia. Our study found a U-shaped relationship (P<0.001) between BMI and biopsy-mediated detection of PCa by crude analysis, which converted to a steady increasing trend after multivariate adjustment. On the contrary, we failed to find any correlation between BMI and HGPCa in a multivariate-adjusted model.

Previous studies have indicated that age and PSA level may obscure the impact of BMI on PCa detection at biopsy [19,20]. In the present study, age was negatively associated with BMI, which was compatible with previous studies [12]. After adjusting for age, the elevated PCa detection risk diminished in the underweight group and was exaggerated in the overweight and obese group, indicating that the contradictory changes in OR might be due to the inverse correlation between age and BMI. Additionally, a significant inverse relationship was observed between BMI and serum PSA [21,22], which may be due to the hypothesis that obese individuals possess lower testosterone levels [19] or larger plasma volumes may mediate a hemodilution effect [23]. In the current study, the PSA levels in the underweight group were lower than those in normal-weight counterparts, which is in agreement with previous studies. In contrast, the PSA levels in the obese group also tended to be higher than those in normal-weight counterparts. After adjusting for different variables, the high OR for PCa detection diminished in the underweight group, but was increased in the overweight and obese groups. Therefore, the relationship between BMI and biopsy-mediated detection of PCa evolved from U-shaped to a steady increasing trend, revealing a biological mechanism that BMI was positively correlated with detection of PCa. Additionally, we found that the risk of being diagnosed with PCa significantly rose as soon as BMI was over 23 kg/m^2^. The threshold of BMI causing an OR shift in our Chinese population was lower than that previously reported in Western counterparts, which might be explained by the higher body fat percentages and the comparatively delayed diagnosis in Asian men [24].

It has been widely reported that a higher BMI provides a favourable biological microenvironment for tumour onset and growth. Men with higher BMI values have also been reported to produce less testosterone, resulting in PCa that is less androgen dependent and consequently more aggressive [25]. Moreover, excessive adiposity might also result in the secretion of various adipokines and inflammatory cytokines, which may promote tumour growth [26]. In addition, obese men tend to show increased levels of insulin and insulin-like growth factor 1 (IGF-1), both of which can inhibit apoptosis and encourage carcinogenesis [27].

Since PSA levels varied greatly across BMI categories, the reliability of serum PSA as a PCa screening tool required tests. In the present study, we found no significant difference in the ability of PSA to predict PCa among different BMI groups. The average AUC of PSA is 0.816 for PCa detection and 0.805 for HGPCa detection, indicating PSA level was still an effective tool to discriminate between cancer and benign diseases at different BMIs.

The result of our study that BMI was positively correlated with PCa detection at biopsy was in line with the researches conducted by Masuda et al [12] and Park et al [16]. While researches by Kobayashi et al [8] and Lee et al [17] brought about total converse conclusion that BMI was negatively correlated with PCa detection. Since the population in China, Japan and Korea shared similar racial background, daily diet and BMI distribution, the contradiction might depend partly on the PSA screening popularity and biopsy cores, which need further investigation.

The present study had some limitations that need to be addressed. First, the study was conducted in a tertiary referral hospital in Shanghai and thus might not represent the whole Chinese population. Second, BMI alone was unable to distinguish fat from muscle, whereas parameters such as waist circumference [28], waist-to-hip ratio [29] and percentage of visceral adipose tissue [30] have recently been shown to be better indicators of obesity. Additionally, the study failed to adjust for additional confounding factors that could possibly be associated with PSA levels, such as the duration of obesity, medication use, comorbidities, daily diet and exercise. Furthermore, the exposure rate of PSA screening for PCa in China is generally lower than that in the USA and Europe. Accordingly, the selection of candidates could have biased the clinical characteristics and, subsequently, the biopsy outcomes.

In conclusion, the present study revealed that obesity, as an independent risk factor, was associated with higher risk of PCa detection in the present Chinese biopsy population, whereas no significant association was detected between obesity and HGPCa.

## Supporting Information

S1 Supporting Information Editorial certificate by American Journal Expert. (PDF)
